# Concentration of total proteins in urine as a non-invasive biomarker of endometriosis

**DOI:** 10.5935/1518-0557.20230028

**Published:** 2023

**Authors:** Alenka Višnić, Dubravko Barišić, Gordana Čanadi Jurešić, Marko Klarić, Tina Sušanj Šepić

**Affiliations:** 1 Clinical Hospital Center Rijeka, Clinic for Gynecology and Obstetrics, Rijeka, Rijeka, Croatia; 2 Special Hospital Radiochirurgia Zagreb, Zagreb, Croatia; 3 Department of Medicinal Chemistry, Biochemistry and Clinical Chemistry, MEF University in Rijeka, Rijeka, Croatia

**Keywords:** endometriosis, infertility, proteins, urine

## Abstract

**Objective:**

The disease in which we observe the invasion and growth of endometrial cells on extrauterine tissues and organs with the creation of a chronic inflammatory state is called endometriosis. It causes infertility and is present in more than 30% of patients with endometriosis. Diagnosis and treatment of the disease is most often delayed for about 8 years after the first symptoms were reported. The symptomatology of endometriosis is varied, and there is no non-invasive way of diagnosis, and this is the reason for the delayed start of treatment. The development of endometriosis activates pathological processes such as the invasion and proliferation of endometriotic cells, the formation of adhesions and the activation of the immune system, which result in increased protein expression. The aim of this research is to compare the concentrations of total proteins in the urine of subjects with endometriosis with those of the control group and possibly identify a biomarker for the diagnosis of endometriosis.

**Methods:**

Prospective urine analysis of 141 patients who were hospitalized and surgically treated at the Clinic for Gynecology and Obstetrics of KBC Rijeka from 08/21/2021 until 07/30/2022. The urine of subjects with endometriosis (N=84) and without endometriosis (n=57) was analyzed.

**Results:**

Total protein in the urine is increased in the urine of subjects with endometriosis, but the total amount of protein does not correlate with the degree of disease progression.

**Conclusions:**

An increase in the level of total proteins in urine in subjects with endometriosis is a possible non-invasive diagnostic biomarker. Patients with endometriosis are grouped after the concentration of total proteins greater than 5000 µg/µl.

## INTRODUCTION

### Definition, history and classification of endometriosis

Endometriosis is a disease which pathogenesis is not fully known. Several factors are responsible for the onset of the disease. However, the etiology of all forms of endometriosis has not been fully explained to date (Bulletti *et al*., 2010). It’s the most common disease in the female population in reproductive age (Broi *et al*., 2019). Endometriosis is the leading cause of infertility and poorer outcomes of assisted reproduction (Marí-Alexandre *et al*., 2018). From the appearance of the first symptoms to the surgical diagnosis, it takes an average of 4 to 11 years. A possible reason is the non-specificity of the symptoms of the disease as well as the impossibility of non-invasive diagnosis of endometriosis (Agarwal *et al*., 2019). Ectopic endometrial tissue interacting with an aberrant immune system leads to the chronic inflammation that characterizes endometriosis (Wang *et al*., 2020). Such an inflamed area causes pain that is not the same in all patients, regardless of the size of the lesions. In addition to pain, endometriosis often causes irregular menstrual bleeding, dyspareunia, dysmenorrhea and, as is known, infertility. It is a chronic debilitating disease that significantly reduces the quality of life of a woman with endometriosis (Parasar *et al*., 2017).

The American Society for Reproductive Medicine has created a classification of endometriosis called the ASRM classification (Metzemaekers *et al*., 2020). The ASRM classification was then supplemented with the ENZIAN classification and is used as the rASRM classification (Lee *et al*., 2021). The pathological processes that occur with the development of endometriosis are invasion, adhesion, proliferation, angiogenesis and impaired immunity. The above-mentioned pathophysiological processes are mutually maintained and lead to disorders of the immune and endocrinological systems and changes in the anatomical relations of the reproductive organs (Burney & Giudice, 2012).

The aim of this research is to compare the concentrations of total proteins in the urine of subjects with endometriosis with those of the control group and possibly identify a biomarker for the diagnosis of endometriosis.

## MATERIALS AND METHODS

For this study, an ethical permit was obtained from the committee of the Rijeka Clinical Hospital Center (Approval number: 003-05/21-1/124). All patients who were included in the research gave their consent by signing an Informed Consent Form. Urine samples were collected from 141 subjects who were treated at the Clinic for Gynecology and Obstetrics of KBC Rijeka for suspected endometriosis and other cystic ovarian diseases. The day before the surgical treatment, the first morning urine was obtained from the subjects. All test subjects were between the ages of 25 and 35, in the proliferative phase of their menstrual cycles. Subjects with kidney disease were excluded from the study, which was determined by urea and creatinine levels. Endometriosis was diagnosed by histopathological diagnosis after surgical treatment. According to the histopathological diagnosis, the urine samples were divided into a group of subjects with endometriosis (n=84) and a control group of subjects without endometriosis (n=57). Subjects with endometriosis were divided into rASRM I-II (n=42) and rASRM III-IV (n=42) subgroups. The subgroups, that is, the stage of the disease, was determined by surgery.

### Pre-analytical preparation of urine

The urine is centrifuged at 3500 rpm for 30 min (Hettich centrifuge, Universal 320/320 R Andreas Hettich GmbH & Co., Tuttlingen, Germany). 10% TCA and 3% DTT were added to the total volume of acetone. The urine supernatant (7.5 ml) is mixed with acetone solution (20 ml). The mixture of urine and acetone (ratio 1:3) was kept at a temperature of -20°C for a minimum of 20 hours to a maximum of 24 hours. After repeated centrifugation and decantation, a protein sediment of urine was obtained, to which a 5 times larger amount of lysis buffer was added, which was a mixture of 2.1g urea, 0.2g CHAPS, 0.7612g thiourea and ddH₂O up to 5 ml. The resulting volume was weighed and 1% protease inhibitors are added to it.

### One-dimensional gel - electrophoresis of urinary proteins

Qualitative determination of protein in urine by one-dimensional electrophoresis was performed to check the quality of the pre-analytical preparation of urine protein precipitates. The method of one-dimensional protein gel electrophoresis gives us information about the purity of the samples, the amount and the molecular weight of the protein. In this study, one-dimensional gel electrophoresis was performed in the classical way. An electrophoresis system (Bio-Rad Hercules, CA, USA) was used.

### Determination of protein concentration in urine by the Bradford method

For proteomic analyzes of proteins in urine, protein concentration was determined using the Bradford reagent, which was prepared in the classic way. The resulting Coomassie Brilliant Blue G-250 solution has a very characteristic blue color and is known as Bradford’s reagent. The standard is bovine serum albumin BSA (from Eng. bovine serum albumin) protein concentration (γ=1 mg/mL). We used a device for colorometric protein determination (Spectrometer Varian Cary 100 and software Cary WinUV, USA).

## RESULTS

Qualitative analysis of protein in urine-protein sediment samples was analyzed with one-dimensional electrophoresis. All urine samples of the endometriosis group and the control group were analyzed using this method. In all analyzed samples, proteins are clearly present and separated. In all samples of urine protein deposits, there is a sufficient amount of proteins of different molecular masses required for further analytical analyses.

### Concentration of total proteins in urine - determined by the Bradford method

Quantitative determination of protein in urine-protein sediment samples was performed using the Bradford method. A statistically significant difference in the concentration of total proteins was recorded between the samples of the group with endometriosis and the samples of the control group. The Wilcoxon test with a confidence level of 95% showed that the concentrations of secreted proteins (µg/µl) in the urine analyzed by the Bradford method in samples with endometriosis (n=84) were statistically significantly higher than in samples of the control group (n=57), *p*<0.05. After the concentration of total proteins in the urine is greater than 5,000 (µg/µl), there is a significant grouping of samples with endometriosis, while at concentrations lower than this value, samples of both groups were grouped.

**Figure 1:**
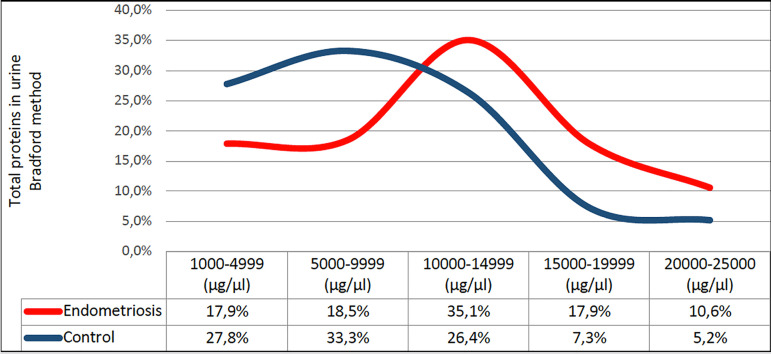
Presentation of the proportion of urine samples in each group depending on the concentration range of total proteins (µg/µl) in the group of subjects with endometriosis and subjects of the control group (Bradford method).

The analysis of samples of endometriosis subgroups rASRM I-II and rASRM III-IV did not show a difference in the concentration of excreted total proteins in the urine. The Wilcoxon test with a confidence level of 95% showed that there was no statistically significant difference in the values of the concentration of secreted proteins (µg/µl) in the urine, analyzed by the Bradford method in subgroups of EME samples - rASRM I-II (n=42) and rASRM III-IV IV (N=42) (Figure 1, Table 1).

**Table 1:** Parameters of the comparison of total protein concentration (µg/µl) in the urine of the group of test subjects with endometriosis and the control group.

TESTED GROUPS	ENDOMETRIOSIS	CONTROL
**NUMBER OF SAMPLES (N)**	84	57
**ANALYTICAL METHOD**	Bradford method	
**MINIMUM (µg/µl)**	2525.89	1094.24
**MAXIMUM (µg/µl)**	25063.29	23809.08
**MEDIAN (µg/µl)**	11487.40	8512.84
**ARITHMETIC MEAN± STANDARD DEVIATION**	11919.78±5384.10	9374.26±5039.74
**STATISTICAL METHOD**	Wilcoxonov test	
**DISTRIBUTION TYPE**	Non-parametric distribution	
**EVALUATION OF THE DIFFERENCE**	2857.4 (CI 95% 1094.7, 4665)	
**STATISTICAL SIGNIFICANCE**	**U=2106 *p*=0.0034**	

## DISCUSSION

In addition to the fact that endometriosis is a disease that affects a number of physiological reactions in the body, it also plays an unquestionable role in the infertility of patients. There are different levels of causes of infertility associated with endometriosis. We often see the disruption of anatomical relationships through the formation of cystic formations, adhesions and fibrosis, which disrupts the tubo-ovarian relationship, reduced ovarian reserves and impaired receptivity of the endometrium (Bulletti *et al*., 2010). The results of this study show a statistically significant association of endometriosis with different types of proteins in the urine. The concentration of total excreted proteins in the first morning urine is statistically significantly higher in the group of samples with endometriosis compared with the control group. Higher concentrations of total excreted proteins in urine were shown in all stages of disease extension according to the rASRM criteria; however, no statistically significant difference was shown in protein concentration in urine depending on the degree of endometriosis extension.

Lessey *et al*. (2015) did not show a difference in the concentration of total proteins in the urine in samples from the group with endometriosis compared to the samples from the control group. Contrary to the results obtained in the aforementioned study, in our investigation we noticed a statistically significantly higher concentration of total proteins in the urine samples with endometriosis. The difference in recorded results was probably due to the different pre-analytical preparation of urine in this study, in contrast to Lessey *et al*. (2015). With the development of endometriosis, a number of proteins and their precursors are secreted with the aim of achieving an equilibrium state in the areas of ectopic invasion and proliferation of endometrial cells (Xiao *et al*., 2017). It is believed that one of the most important roles in the etiopathogenesis of endometriosis is played by the pro-inflammatory activated immune system. In the peritoneal space, immune factors are secreted in an increased amount, they modulate the immune response and open space for extrauterine implantation and proliferation of endometrial cells.

Despite numerous studies, it cannot be asserted whether the pro-inflammatory activated immune system is the cause of endometriosis, or a factor in its progression (Ahn *et al*., 2015). Peritoneal fluid in endometriosis is rich in pro-inflammatory cells. The resulting anaphylatoxins stimulate peritoneal macrophages and mast cells to increase secretion of cytokines and histamine (Agostinis *et al*., 2021). Proteases hydrolyze peptide bonds and break down protein chains into smaller fragments. With such reactions, proteins lose their controlled primary activity and cause the breakdown of the extracellular space, which opens up space for the development of endometriosis (Bałkowiec *et al.,* 2018). Protease inhibitors have the opposite effect and suppress their activity by binding to the receptors of enzymatically active proteins (Porter *et al*., 2016).

In ectopic endometrial cells, there is an inadequate relationship between apoptosis factors and those that promote cell growth (Li *et al*., 2020). In addition to the uneven relationship between growth factors and apoptosis, ectopic endometrial cells are very resistant to apoptosis. Altered cells of the peritoneum enable better efficiency of proteins that promote proliferation and reduced function of apoptotic proteins (Renner *et al*., 2015). These are just some of the processes that occur during the development of endometriosis. The body continuously tries to achieve homeostasis. Since homeostasis in endometriosis is impossible to achieve, the increased production of all proteins occurs. Both those that serve the proliferation of endometriotic cells, as well as those proteins that try to suppress such processes (Zhang *et al*., 2006). The result is an increased production of proteins that are eventually excreted in the urine and it is possible to detect them and determine their concentration. Proliferation, invasion and apoptosis of ectopic endometrial cells with the formation of adhesions in a chronically inflammatory changed medium is the basis of endometriosis (Agostinis *et al*., 2021; Ferrero, 2019). All the pathological processes that occur during this have as a result the increased production of proteins, and it is not surprising that we can detect them in the urine (Grande *et al*., 2020).

However, although we notice an increased concentration of protein in the urine in subjects with endometriosis, the progression of the disease does not lead to even greater protein production. It is possible that an increase in the degree of progression of endometriosis leads to a kind of exhaustion of the organism, which is manifested by a reduced possibility of further expression of proteins aimed at establishing homeostasis. Such a condition opens the way for the development of endometriosis with a severe clinical picture.

## CONCLUSION

Endometriosis is a disease for which it is necessary to find a specific non-invasive biomarker so that treatment can be started at the very beginning of the disease development. By postponing diagnosis, and thus treatment, the processes that take place in the development of endometriosis, which among other things has infertility as a reason for its progress. The concentration of total proteins in the urine could be a possible biomarker for endometriosis diagnosis.
